# 4-(4-Bromo­phen­yl)-3-methyl-1-phenyl-6,7-dihydro-1*H*-pyrazolo­[3,4-*b*]thieno[2,3-*e*]pyridine 5,5-dioxide

**DOI:** 10.1107/S1600536811045697

**Published:** 2011-11-05

**Authors:** Tuanjie Li, Honghong Zhang

**Affiliations:** a School of Chemistry and Engineering, Jiangsu Key Laboratory of Green Synthetic Chemistry for Functional Materials, Xuzhou Normal University, Xuzhou, Jiangsu 221116, People’s Republic of China

## Abstract

In the title compound, C_21_H_16_BrN_3_O_2_S, the pyrazole and pyridine rings are nearly coplanar, the dihedral angle between their planes being 3.17 (14)°. The 2,3-dihydro­thio­phene ring adopts an envelope conformation. The 4-bromo­phen­yl/pyridine ring and phen­yl/pyrazole rings form dihedral angles of 60.06 (9) and 33.49 (11)°, respectively. There is an intra­molecular C—H⋯N hydrogen bond. The crystal packing is stabilized by inter­molecular C—H⋯O hydrogen bonding and C—H⋯π inter­actions.

## Related literature

For the bioactivity of thienopyridine derivatives, see: Goerlitzer *et al.* (2004 ▶, 2000 ▶); Kamel *et al.* (2003 ▶). For the preparation of the title compound, see: Shi & Yang (2011 ▶).
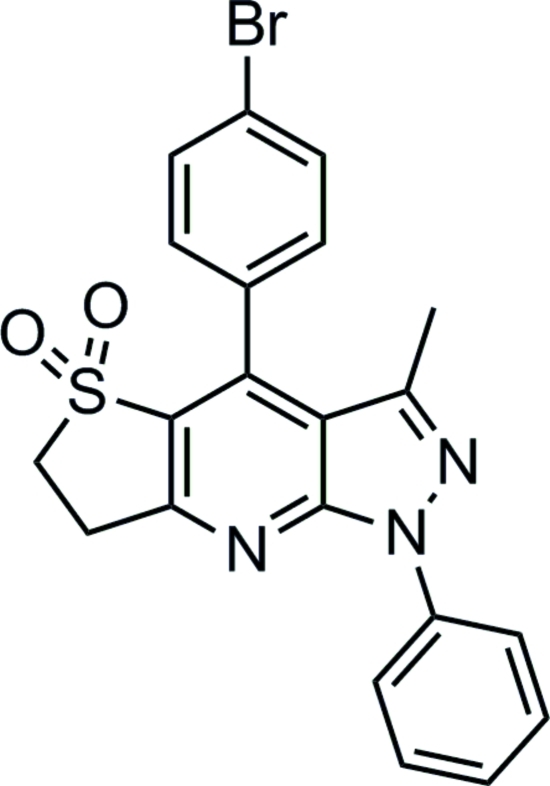


## Experimental

### 

#### Crystal data

C_21_H_16_BrN_3_O_2_S
*M* *_r_* = 454.34
Triclinic, 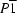

*a* = 9.881 (3) Å
*b* = 9.904 (3) Å
*c* = 11.333 (4) Å
α = 108.642 (1)°
β = 102.000 (4)°
γ = 107.346 (3)°
*V* = 945.1 (5) Å^3^
*Z* = 2
Mo *K*α radiation
μ = 2.31 mm^−1^
*T* = 113 K
0.24 × 0.22 × 0.16 mm

#### Data collection

Rigaku Saturn CCD area-detector diffractometer
Absorption correction: multi-scan (*CrystalClear*; Rigaku/MSC, 2002) ▶ *T*_min_ = 0.607, *T*_max_ = 0.709
10810 measured reflections
4429 independent reflections
2695 reflections with *I* > 2σ(*I*)
*R*_int_ = 0.041

#### Refinement

*R*[*F* ^2^ > 2σ(*F* ^2^)] = 0.036
*wR*(*F* ^2^) = 0.096
*S* = 0.98
4429 reflections
254 parameters
H-atom parameters constrained
Δρ_max_ = 1.14 e Å^−3^
Δρ_min_ = −0.58 e Å^−3^

### 

Data collection: *CrystalClear* (Rigaku/MSC, 2002 ▶); cell refinement: *CrystalClear*; data reduction: *CrystalClear*; program(s) used to solve structure: *SHELXS97* (Sheldrick, 2008 ▶); program(s) used to refine structure: *SHELXL97* (Sheldrick, 2008 ▶); molecular graphics: *SHELXTL* (Sheldrick, 2008 ▶); software used to prepare material for publication: *SHELXTL*.

## Supplementary Material

Crystal structure: contains datablock(s) I, global. DOI: 10.1107/S1600536811045697/hg5128sup1.cif

Structure factors: contains datablock(s) I. DOI: 10.1107/S1600536811045697/hg5128Isup2.hkl

Supplementary material file. DOI: 10.1107/S1600536811045697/hg5128Isup3.cml

Additional supplementary materials:  crystallographic information; 3D view; checkCIF report

## Figures and Tables

**Table 1:** Hydrogen-bond geometry (Å, °)

*D*—H⋯*A*	*D*—H	H⋯*A*	*D*⋯*A*	*D*—H⋯*A*
C3—H3⋯*Cg*^i^	0.95	2.84	3.235 (3)	106
C5—H5⋯O1^ii^	0.95	2.48	3.239 (3)	136
C19—H19⋯O1^iii^	0.95	2.57	3.514 (4)	174
C21—H21⋯N1	0.95	2.56	3.065 (3)	114

Symmetry codes: (i) 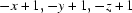; (ii) 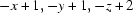; (iii) 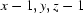.

**Fig. 1.**
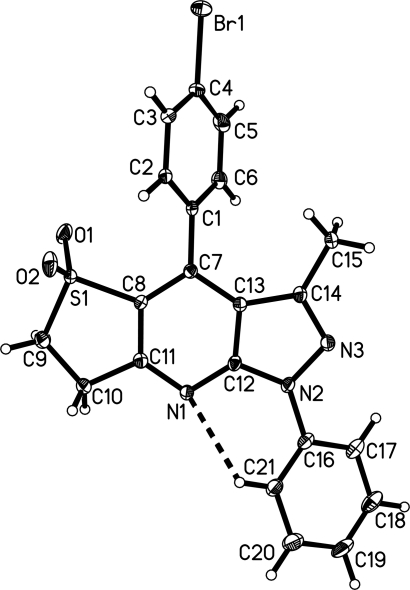
The structure of (I), showing 30% probability displacement ellipsoids and the atom-numbering scheme, hydrogen bond represented by the dashed line. Cg is the centroid of the ring of C16/C17/C18/C19/C20/C21.

**Fig. 2.**
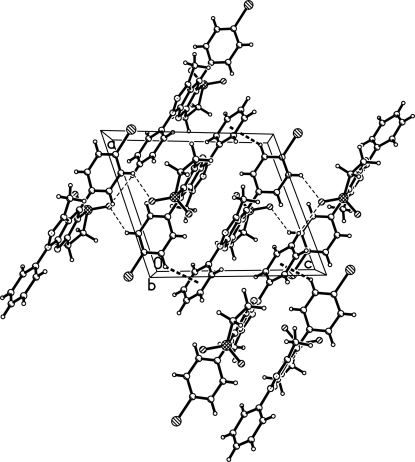
The packing diagram of (I), hydrogen bond represented by the dashed line.

## supplementary crystallographic information

### Comment

Some synthetic thienopyridine compounds exhibit antimalarial activities (Goerlitzer *et al.* 2004) and act as gyrase inhibitors (Goerlitzer *et al.* 2000). Recently, A-312110, a thienopyridine derivative was also reported as a potent KATP channel opener (Kamel *et al.* 2003). These reports inspired us to study the relationship between their structures and activities. During the synthesis of thienopyridine derivatives, the title compound, (I) was isolated and its structure was determined by X-ray diffraction. In the molecular structure (Fig. 1), the pyrazole and pyridine rings adopt planar conformations, with RMS of 0.0035 Å and 0.0170 Å, respectively. The largest deviation of the two rings are 0.005 (1) Å(N2) and 0.0325 (2) Å(C7), respectively. They are nearly coplanar, since the dihedral angle between them is 3.17 (14) °. The 2,3-dihydrothiophene ring adopts an envelope conformation. The distance between atom C9 and the plane of C10/C11/C8/S1 is 0.322 (4) Å. Besides, the 4-bromophenyl and pyridine ring, phenyl and pyrazole ring forms dihedral angles of 60.06 (9)° and 33.49 (11)° respectively. There is an intramolecular C—H···O hydrogen bond. In addition, the crystal packing is stabilized by intermolecular C—H···O hydrogen bond and C—H··· π interactions (Fig. 2).

### Experimental

The title compound was synthesized according to the procedure (Shi *et al.* 2011). A dry 50 ml flask was charged with 4-bromobenzaldehyde (1 mmol), dihydrothiophen-3(2*H*)-one 1,1-dioxide (1 mmol), 5-amino-3-methyl-1-phenyl-pyrazole (1 mmol), and ionic liquid [bmim]Br (2 ml). The mixture was stirred at 363 K for 1.5 h to complete the reaction (monitored by TLC), then 5 ml water was added. The solid was filtered off and washed with water. The crude product was purified by recrystallization from the mixture of DMF and ethanol to give pure product.The recrystallization gave single-crystals suitable for X-ray diffraction.

### Refinement

The H atoms were placed in calculated positions, with C—H = 0.95 Å (aromatic), 0.98Å (methyl) or 0.99Å (methylene)and included in the final cycles of refinement using a riding model, with U_iso_(H) = 1.2U_eq_C(aromatic, methylene) and 1.5U_eq_C(methyl).

### Crystal data

**Table:**

C_21_H_16_BrN_3_O_2_S	*Z* = 2
*M**_r_* = 454.34	*F*(000) = 460
Triclinic, *P*1	*D*_x_ = 1.597 Mg m^−^^3^
Hall symbol: -P 1	Mo *K*α radiation, λ = 0.71073 Å
*a* = 9.881 (3) Å	Cell parameters from 3340 reflections
*b* = 9.904 (3) Å	θ = 2.0–27.9°
*c* = 11.333 (4) Å	µ = 2.31 mm^−^^1^
α = 108.642 (1)°	*T* = 113 K
β = 102.000 (4)°	Prism, colorless
γ = 107.346 (3)°	0.24 × 0.22 × 0.16 mm
*V* = 945.1 (5) Å^3^	

### Data collection

**Table:**

Rigaku Saturn CCD area-detector diffractometer	4429 independent reflections
Radiation source: rotating anode	2695 reflections with *I* > 2σ(*I*)
multilayer	*R*_int_ = 0.041
Detector resolution: 14.63 pixels mm^-1^	θ_max_ = 27.9°, θ_min_ = 2.0°
ω and φ scans	*h* = −11→12
Absorption correction: multi-scan (*CrystalClear*; Rigaku/MSC, 2002)	*k* = −13→13
*T*_min_ = 0.607, *T*_max_ = 0.709	*l* = −14→14
10810 measured reflections	

### Refinement

**Table:**

Refinement on *F*^2^	Primary atom site location: structure-invariant direct methods
Least-squares matrix: full	Secondary atom site location: difference Fourier map
*R*[*F*^2^ > 2σ(*F*^2^)] = 0.036	Hydrogen site location: inferred from neighbouring sites
*wR*(*F*^2^) = 0.096	H-atom parameters constrained
*S* = 0.98	*w* = 1/[σ^2^(*F*_o_^2^) + (0.0437*P*)^2^] where *P* = (*F*_o_^2^ + 2*F*_c_^2^)/3
4429 reflections	(Δ/σ)_max_ = 0.001
254 parameters	Δρ_max_ = 1.14 e Å^−^^3^
0 restraints	Δρ_min_ = −0.58 e Å^−^^3^

### Special details

**Table:**

Geometry. All e.s.d.'s (except the e.s.d. in the dihedral angle between two l.s. planes) are estimated using the full covariance matrix. The cell e.s.d.'s are taken into account individually in the estimation of e.s.d.'s in distances, angles and torsion angles; correlations between e.s.d.'s in cell parameters are only used when they are defined by crystal symmetry. An approximate (isotropic) treatment of cell e.s.d.'s is used for estimating e.s.d.'s involving l.s. planes.
Refinement. Refinement of *F*^2^ against ALL reflections. The weighted *R*-factor *wR* and goodness of fit *S* are based on *F*^2^, conventional *R*-factors *R* are based on *F*, with *F* set to zero for negative *F*^2^. The threshold expression of *F*^2^ > σ(*F*^2^) is used only for calculating *R*-factors(gt) *etc*. and is not relevant to the choice of reflections for refinement. *R*-factors based on *F*^2^ are statistically about twice as large as those based on *F*, and *R*- factors based on ALL data will be even larger.

### Fractional atomic coordinates and isotropic or equivalent isotropic displacement parameters (Å^2^)

**Table:**

	*x*	*y*	*z*	*U*_iso_*/*U*_eq_	
Br1	1.02598 (4)	0.80211 (4)	1.16776 (3)	0.04812 (13)	
S1	0.45768 (7)	0.09317 (7)	0.71274 (6)	0.02120 (15)	
O1	0.4863 (2)	0.1482 (2)	0.85218 (18)	0.0380 (5)	
O2	0.5755 (2)	0.0686 (2)	0.6663 (2)	0.0372 (5)	
N1	0.1738 (2)	0.1677 (2)	0.4704 (2)	0.0220 (5)	
N2	0.1926 (2)	0.4054 (2)	0.4460 (2)	0.0233 (5)	
N3	0.2917 (2)	0.5588 (2)	0.5057 (2)	0.0240 (5)	
C1	0.5936 (3)	0.4707 (3)	0.8034 (2)	0.0190 (5)	
C2	0.7289 (3)	0.4526 (3)	0.8108 (2)	0.0204 (5)	
H2	0.7331	0.3712	0.7411	0.025*	
C3	0.8563 (3)	0.5509 (3)	0.9177 (2)	0.0243 (6)	
H3	0.9490	0.5395	0.9206	0.029*	
C4	0.8497 (3)	0.6665 (3)	1.0212 (2)	0.0273 (6)	
C5	0.7152 (3)	0.6852 (3)	1.0175 (3)	0.0318 (7)	
H5	0.7111	0.7644	1.0892	0.038*	
C6	0.5877 (3)	0.5877 (3)	0.9089 (3)	0.0270 (6)	
H6	0.4954	0.6001	0.9057	0.032*	
C7	0.4559 (3)	0.3682 (3)	0.6872 (2)	0.0193 (5)	
C8	0.3902 (3)	0.2086 (3)	0.6455 (2)	0.0183 (5)	
C9	0.2875 (3)	−0.0719 (3)	0.6279 (3)	0.0416 (8)	
H9A	0.3088	−0.1659	0.5938	0.050*	
H9B	0.2307	−0.0839	0.6888	0.050*	
C10	0.1963 (3)	−0.0532 (3)	0.5156 (3)	0.0291 (6)	
H10A	0.2062	−0.1134	0.4316	0.035*	
H10B	0.0887	−0.0919	0.5076	0.035*	
C11	0.2532 (3)	0.1152 (3)	0.5425 (2)	0.0210 (5)	
C12	0.2421 (3)	0.3213 (3)	0.5059 (2)	0.0205 (5)	
C13	0.3777 (3)	0.4255 (3)	0.6098 (2)	0.0206 (5)	
C14	0.4020 (3)	0.5728 (3)	0.6035 (2)	0.0214 (5)	
C15	0.5315 (3)	0.7246 (3)	0.6841 (3)	0.0267 (6)	
H15A	0.5434	0.7867	0.6320	0.040*	
H15B	0.6236	0.7069	0.7099	0.040*	
H15C	0.5127	0.7800	0.7636	0.040*	
C16	0.0596 (3)	0.3565 (3)	0.3378 (3)	0.0260 (6)	
C17	−0.0100 (3)	0.4602 (4)	0.3356 (3)	0.0310 (7)	
H17	0.0300	0.5609	0.4049	0.037*	
C18	−0.1381 (3)	0.4129 (4)	0.2301 (3)	0.0393 (8)	
H18	−0.1857	0.4828	0.2273	0.047*	
C19	−0.1981 (3)	0.2683 (4)	0.1296 (3)	0.0446 (8)	
H19	−0.2872	0.2380	0.0589	0.054*	
C20	−0.1277 (3)	0.1653 (4)	0.1315 (3)	0.0433 (8)	
H20	−0.1680	0.0651	0.0616	0.052*	
C21	0.0016 (3)	0.2105 (3)	0.2362 (3)	0.0337 (7)	
H21	0.0502	0.1412	0.2379	0.040*	

### Atomic displacement parameters (Å^2^)

**Table:**

	*U*^11^	*U*^22^	*U*^33^	*U*^12^	*U*^13^	*U*^23^
Br1	0.0535 (2)	0.02996 (18)	0.02916 (18)	0.00753 (16)	−0.01463 (14)	−0.00030 (13)
S1	0.0202 (3)	0.0170 (3)	0.0251 (3)	0.0074 (3)	0.0038 (3)	0.0093 (3)
O1	0.0592 (14)	0.0385 (12)	0.0241 (10)	0.0289 (11)	0.0091 (10)	0.0163 (9)
O2	0.0347 (12)	0.0336 (12)	0.0642 (15)	0.0229 (10)	0.0272 (11)	0.0300 (11)
N1	0.0198 (12)	0.0196 (11)	0.0260 (12)	0.0070 (9)	0.0047 (9)	0.0112 (9)
N2	0.0201 (12)	0.0233 (12)	0.0277 (12)	0.0089 (10)	0.0036 (9)	0.0141 (10)
N3	0.0222 (12)	0.0209 (12)	0.0294 (12)	0.0080 (10)	0.0067 (10)	0.0124 (10)
C1	0.0213 (14)	0.0160 (12)	0.0218 (13)	0.0071 (11)	0.0065 (10)	0.0109 (10)
C2	0.0237 (14)	0.0172 (13)	0.0193 (13)	0.0087 (11)	0.0046 (10)	0.0072 (10)
C3	0.0228 (14)	0.0219 (14)	0.0245 (14)	0.0077 (12)	0.0018 (11)	0.0097 (11)
C4	0.0302 (16)	0.0198 (14)	0.0196 (13)	0.0047 (12)	−0.0028 (11)	0.0048 (11)
C5	0.0472 (19)	0.0215 (14)	0.0224 (14)	0.0138 (14)	0.0121 (13)	0.0032 (11)
C6	0.0306 (16)	0.0253 (14)	0.0293 (15)	0.0153 (13)	0.0128 (12)	0.0107 (12)
C7	0.0173 (13)	0.0190 (13)	0.0225 (13)	0.0085 (11)	0.0061 (10)	0.0086 (11)
C8	0.0186 (13)	0.0196 (13)	0.0192 (12)	0.0092 (11)	0.0063 (10)	0.0095 (10)
C9	0.0330 (18)	0.0248 (16)	0.053 (2)	0.0000 (14)	−0.0055 (14)	0.0215 (15)
C10	0.0248 (15)	0.0203 (14)	0.0343 (16)	0.0050 (12)	0.0011 (12)	0.0109 (12)
C11	0.0201 (14)	0.0207 (13)	0.0234 (13)	0.0081 (11)	0.0084 (11)	0.0096 (11)
C12	0.0168 (13)	0.0219 (13)	0.0272 (14)	0.0092 (11)	0.0075 (11)	0.0136 (11)
C13	0.0204 (14)	0.0187 (13)	0.0228 (13)	0.0078 (11)	0.0060 (11)	0.0094 (11)
C14	0.0221 (14)	0.0201 (13)	0.0257 (14)	0.0103 (12)	0.0097 (11)	0.0108 (11)
C15	0.0272 (15)	0.0189 (13)	0.0321 (15)	0.0081 (12)	0.0050 (12)	0.0122 (12)
C16	0.0170 (14)	0.0354 (16)	0.0294 (15)	0.0070 (12)	0.0064 (11)	0.0218 (13)
C17	0.0300 (17)	0.0483 (19)	0.0311 (15)	0.0235 (15)	0.0155 (13)	0.0253 (14)
C18	0.0275 (17)	0.069 (2)	0.0427 (19)	0.0285 (18)	0.0170 (15)	0.0368 (18)
C19	0.0242 (17)	0.067 (2)	0.0415 (19)	0.0077 (16)	−0.0007 (13)	0.0368 (18)
C20	0.0358 (18)	0.046 (2)	0.0355 (17)	0.0008 (16)	−0.0005 (14)	0.0236 (16)
C21	0.0294 (16)	0.0334 (17)	0.0348 (16)	0.0070 (14)	0.0014 (13)	0.0205 (14)

### Geometric parameters (Å, °)

**Table:**

Br1—C4	1.890 (3)	C8—C11	1.399 (3)
S1—O2	1.4261 (19)	C9—C10	1.497 (4)
S1—O1	1.4304 (19)	C9—H9A	0.9900
S1—C9	1.763 (3)	C9—H9B	0.9900
S1—C8	1.770 (2)	C10—C11	1.495 (4)
N1—C11	1.342 (3)	C10—H10A	0.9900
N1—C12	1.347 (3)	C10—H10B	0.9900
N2—C12	1.364 (3)	C12—C13	1.411 (3)
N2—N3	1.381 (3)	C13—C14	1.434 (3)
N2—C16	1.427 (3)	C14—C15	1.490 (3)
N3—C14	1.322 (3)	C15—H15A	0.9800
C1—C2	1.390 (3)	C15—H15B	0.9800
C1—C6	1.400 (3)	C15—H15C	0.9800
C1—C7	1.481 (3)	C16—C21	1.383 (4)
C2—C3	1.372 (3)	C16—C17	1.400 (4)
C2—H2	0.9500	C17—C18	1.382 (4)
C3—C4	1.381 (4)	C17—H17	0.9500
C3—H3	0.9500	C18—C19	1.367 (5)
C4—C5	1.389 (4)	C18—H18	0.9500
C5—C6	1.380 (4)	C19—C20	1.399 (4)
C5—H5	0.9500	C19—H19	0.9500
C6—H6	0.9500	C20—C21	1.389 (4)
C7—C8	1.388 (3)	C20—H20	0.9500
C7—C13	1.409 (3)	C21—H21	0.9500
			
O2—S1—O1	117.48 (13)	C11—C10—H10A	110.1
O2—S1—C9	111.19 (15)	C9—C10—H10A	110.1
O1—S1—C9	110.26 (14)	C11—C10—H10B	110.1
O2—S1—C8	109.80 (11)	C9—C10—H10B	110.1
O1—S1—C8	111.74 (11)	H10A—C10—H10B	108.4
C9—S1—C8	93.92 (13)	N1—C11—C8	124.4 (2)
C11—N1—C12	112.6 (2)	N1—C11—C10	120.2 (2)
C12—N2—N3	110.6 (2)	C8—C11—C10	115.4 (2)
C12—N2—C16	129.5 (2)	N1—C12—N2	125.9 (2)
N3—N2—C16	119.9 (2)	N1—C12—C13	127.2 (2)
C14—N3—N2	107.4 (2)	N2—C12—C13	106.9 (2)
C2—C1—C6	119.0 (2)	C7—C13—C12	119.0 (2)
C2—C1—C7	121.4 (2)	C7—C13—C14	136.0 (2)
C6—C1—C7	119.5 (2)	C12—C13—C14	104.9 (2)
C3—C2—C1	120.7 (2)	N3—C14—C13	110.2 (2)
C3—C2—H2	119.6	N3—C14—C15	120.5 (2)
C1—C2—H2	119.6	C13—C14—C15	129.3 (2)
C2—C3—C4	119.8 (2)	C14—C15—H15A	109.5
C2—C3—H3	120.1	C14—C15—H15B	109.5
C4—C3—H3	120.1	H15A—C15—H15B	109.5
C3—C4—C5	120.6 (2)	C14—C15—H15C	109.5
C3—C4—Br1	119.6 (2)	H15A—C15—H15C	109.5
C5—C4—Br1	119.8 (2)	H15B—C15—H15C	109.5
C6—C5—C4	119.5 (2)	C21—C16—C17	120.6 (3)
C6—C5—H5	120.3	C21—C16—N2	120.8 (2)
C4—C5—H5	120.3	C17—C16—N2	118.6 (3)
C5—C6—C1	120.3 (2)	C18—C17—C16	118.5 (3)
C5—C6—H6	119.9	C18—C17—H17	120.8
C1—C6—H6	119.9	C16—C17—H17	120.8
C8—C7—C13	113.7 (2)	C19—C18—C17	121.8 (3)
C8—C7—C1	123.8 (2)	C19—C18—H18	119.1
C13—C7—C1	122.5 (2)	C17—C18—H18	119.1
C7—C8—C11	122.9 (2)	C18—C19—C20	119.7 (3)
C7—C8—S1	127.27 (19)	C18—C19—H19	120.2
C11—C8—S1	109.75 (18)	C20—C19—H19	120.2
C10—C9—S1	108.94 (19)	C21—C20—C19	119.6 (3)
C10—C9—H9A	109.9	C21—C20—H20	120.2
S1—C9—H9A	109.9	C19—C20—H20	120.2
C10—C9—H9B	109.9	C16—C21—C20	119.9 (3)
S1—C9—H9B	109.9	C16—C21—H21	120.1
H9A—C9—H9B	108.3	C20—C21—H21	120.1
C11—C10—C9	108.2 (2)		
			
C12—N2—N3—C14	−0.8 (3)	S1—C8—C11—C10	1.0 (3)
C16—N2—N3—C14	178.7 (2)	C9—C10—C11—N1	−167.5 (2)
C6—C1—C2—C3	−2.2 (4)	C9—C10—C11—C8	12.2 (3)
C7—C1—C2—C3	178.4 (2)	C11—N1—C12—N2	176.3 (2)
C1—C2—C3—C4	1.9 (4)	C11—N1—C12—C13	−3.1 (3)
C2—C3—C4—C5	−0.6 (4)	N3—N2—C12—N1	−178.5 (2)
C2—C3—C4—Br1	−179.70 (18)	C16—N2—C12—N1	2.0 (4)
C3—C4—C5—C6	−0.4 (4)	N3—N2—C12—C13	0.9 (3)
Br1—C4—C5—C6	178.66 (19)	C16—N2—C12—C13	−178.5 (2)
C4—C5—C6—C1	0.1 (4)	C8—C7—C13—C12	3.0 (3)
C2—C1—C6—C5	1.1 (4)	C1—C7—C13—C12	−176.0 (2)
C7—C1—C6—C5	−179.5 (2)	C8—C7—C13—C14	−174.1 (3)
C2—C1—C7—C8	59.7 (3)	C1—C7—C13—C14	7.0 (4)
C6—C1—C7—C8	−119.7 (3)	N1—C12—C13—C7	0.9 (4)
C2—C1—C7—C13	−121.5 (3)	N2—C12—C13—C7	−178.6 (2)
C6—C1—C7—C13	59.2 (3)	N1—C12—C13—C14	178.8 (2)
C13—C7—C8—C11	−4.6 (3)	N2—C12—C13—C14	−0.7 (3)
C1—C7—C8—C11	174.4 (2)	N2—N3—C14—C13	0.3 (3)
C13—C7—C8—S1	177.58 (18)	N2—N3—C14—C15	177.3 (2)
C1—C7—C8—S1	−3.5 (3)	C7—C13—C14—N3	177.6 (3)
O2—S1—C8—C7	−78.9 (2)	C12—C13—C14—N3	0.2 (3)
O1—S1—C8—C7	53.3 (2)	C7—C13—C14—C15	0.9 (5)
C9—S1—C8—C7	166.9 (2)	C12—C13—C14—C15	−176.5 (2)
O2—S1—C8—C11	103.01 (19)	C12—N2—C16—C21	−34.4 (4)
O1—S1—C8—C11	−124.82 (18)	N3—N2—C16—C21	146.2 (2)
C9—S1—C8—C11	−11.2 (2)	C12—N2—C16—C17	146.6 (2)
O2—S1—C9—C10	−95.0 (2)	N3—N2—C16—C17	−32.9 (3)
O1—S1—C9—C10	132.9 (2)	C21—C16—C17—C18	0.4 (4)
C8—S1—C9—C10	18.0 (2)	N2—C16—C17—C18	179.5 (2)
S1—C9—C10—C11	−19.6 (3)	C16—C17—C18—C19	0.5 (4)
C12—N1—C11—C8	1.4 (3)	C17—C18—C19—C20	−1.0 (5)
C12—N1—C11—C10	−178.9 (2)	C18—C19—C20—C21	0.7 (4)
C7—C8—C11—N1	2.5 (4)	C17—C16—C21—C20	−0.8 (4)
S1—C8—C11—N1	−179.29 (19)	N2—C16—C21—C20	−179.8 (2)
C7—C8—C11—C10	−177.2 (2)	C19—C20—C21—C16	0.2 (4)

### Hydrogen-bond geometry (Å, °)

**Table:**

*D*—H···*A*	*D*—H	H···*A*	*D*···*A*	*D*—H···*A*
C3—H3···Cg^i^	0.95	2.84	3.235 (3)	106.
C5—H5···O1^ii^	0.95	2.48	3.239 (3)	136.
C19—H19···O1^iii^	0.95	2.57	3.514 (4)	174.
C21—H21···N1	0.95	2.56	3.065 (3)	114.

Symmetry codes: (i) −*x*+1, −*y*+1, −*z*+1; (ii) −*x*+1, −*y*+1, −*z*+2; (iii) *x*−1, *y*, *z*−1.

## References

[bb1] Goerlitzer, K., Kramer, C. & Boyle, C. (2000). *Pharmazie*, **55**, 595–600. 10989837

[bb2] Goerlitzer, K., Meyer, H., Walter, R. D., Jomaa, H. & Wiesner, J. (2004). *Pharmazie*, **59**, 506–512. 15297995

[bb3] Kamel, M. M., El-Deen, E. M. M. & Abdou, W. A. M. (2003). *Bull. Fac. Pharm.* **41**, 197–206.

[bb4] Rigaku/MSC (2002). *CrystalClear* Rigaku/MSC Inc., The Woodlands, Texas, USA.

[bb5] Sheldrick, G. M. (2008). *Acta Cryst.* A**64**, 112–122. 10.1107/S0108767307043930 18156677

[bb6] Shi, D.-Q. & Yang, F. (2011). *J. Heterocycl. Chem.* **48**, 308–311.

